# Advanced Visualization of Peroneal Artery Perforators Prior to Autologous Transplantation in Head and Neck Surgery by Dual-Energy CTA and Semiautomatic Vessel Unfolding

**DOI:** 10.3390/jcm15145698

**Published:** 2026-07-21

**Authors:** Marco Wiesmueller, Konstantin Hellwig, Maximilian Hinsen, Markus Kopp, Claudius Sebastian Mathy, Tobias Moest, Marco Kesting, Michael Uder, Matthias Stefan May

**Affiliations:** 1 Institute of Radiology, University Hospital Erlangen, Friedrich-Alexander-Universität Erlangen-Nürnberg (FAU), 91054 Erlangen, Germany; konstantin.hellwig@uk-erlangen.de (K.H.); maximilian.hinsen@uk-erlangen.de (M.H.); markus.kopp@uk-erlangen.de (M.K.); claudius.mathy@uk-erlangen.de (C.S.M.); michael.uder@uk-erlangen.de (M.U.); matthias.may@uk-erlangen.de (M.S.M.); 2 Image Science Institute, University Hospital Erlangen, Friedrich-Alexander-Universität Erlangen-Nürnberg (FAU), 91054 Erlangen, Germany; 3 Department of Oral and Maxillofacial Surgery, Friedrich-Alexander-Universität Erlangen-Nürnberg (FAU), 91054 Erlangen, Germany; tobias.moest@uk-erlangen.de (T.M.); marco.kesting@uk-erlangen.de (M.K.)

**Keywords:** peroneal perforator arteries, osteomyocutaneous fibular flap surgery, computed tomography angiography (CTA), dual-energy, vessel unfolding reconstruction algorithm (VUR)

## Abstract

**Background/Objectives**: Accurate visualization of peroneal perforator vessels prior to autologous transplantation of osteomyocutaneous fibular flap is essential for surgical success. Our aim was to improve and simplify pre-surgical diagnostics of peroneal perforators using a dedicated dual-energy Computed Tomography Angiography (CTA) protocol and semiautomatic Vessel Unfolding Reconstruction algorithm (VUR). **Methods**: CTA of both lower legs was performed in 22 patients using dual-energy acquisitions from a third-generation dual-source CT scanner and a high iodine flux (7 mL/s, 350 mg/mL). Low-energy virtual monoenergetic reconstructions (40 keV) were automatically reconstructed from the scanner and used for centerline labeling of the peroneal arteries and their perforators on a post-processing console using a dedicated vascular workflow. Separate segmentation and curved multiplanar reconstructions (MPRs) of each identified peroneal perforator vessel were regarded as the gold standard. Traditional visualization techniques of the entire volume like thin-slice maximum intensity projections (MIPs) or the volume rendering technique (VRT) were compared to a new VUR algorithm that aimed to adjust the visualization plane to the course of the vessels. The identified numbers and lengths of the perforator arteries were compared between curved MPRs, thin-slice MIPs in oblique coronal orientation, posterior-view VRT and coronal VUR of each lower leg. **Results**: The VUR algorithm was feasible in all patients and the same quantity of peroneal perforator vessels could be detected in comparison to the gold standard. The mean number of perforator vessels per lower leg was 2.6. Mean perforator length in VUR was slightly shorter by 1.6% and did not significantly differ from curved MPRs (*p* = 0.54), whereas length values from oblique coronal MIP and VRT reconstructions were significantly shorter (both *p* < 0.001). **Conclusions**: The combination of virtual monoenergetic reconstructions and the VUR algorithm enables comprehensive and precise depiction of small peroneal perforator vessels prior to autologous fibular flap transplantation, representing a diagnostic tool comparable to traditional visualization methods.

## 1. Introduction

Microvascular techniques harvesting autologous osteomyocutaneous fibular flaps for mandibular reconstruction have been developed and continuously refined since the 1980s [1,2]. To date, osteomyocutaneous fibular flaps represent the reconstructive gold standard for composite jaw defects [2,3] allowing reconstruction of both extensive osseous and soft tissue defects [4]. Various different surgical methods have been described in the literature [2]. The currently favored surgical procedure, offering high success rates and minimal donor-site morbidity, is autologous transplantation from the lateral limb side [5,6]. Nevertheless, post-surgical complications, when they occur, can be devastating. The most severe of these is complete or partial loss of the skin paddle [7,8,9]. Previous studies show that fibular graft failure is mainly due to poor vessel supply by its perforator arteries [10]. Fibular perforator arteries originate from the fibular artery and traverse through the posterior peroneal intermuscular septum supplying the lateral subcutaneous and cutaneous tissue [11]. 

Pre-surgical imaging and characterization of peroneal perforator arteries are essential for surgical success and help minimize complications [10,12]. Peroneal perforators typically measure less than 2 mm [13]. Their small caliber, oblique course and proximity to the fibular bone hinders simple detection in the clinical routine. Hence, a dedicated and reliable imaging method is needed. Various imaging methods have been previously described, ranging from ultrasound examinations to time-resolved Magnetic Resonance Angiography of the lower limbs [14,15,16,17,18]. Computed Tomography Angiography (CTA) is a quick, non-invasive method used to visualize the vascular system after intravenous administration of an iodine contrast agent [19]. State-of-the-art CT scanners provide high spatial resolution in the sub-millimeter range, and dedicated post-processing software enables three-dimensional visualization of the acquired datasets [20]. However, in the lower limbs, vascular contrast may be compromised due to the long distance from the heart and the small caliber of the vessels. Dual-energy CT (DECT) has the potential to further exploit iodine contrast and may help to overcome this problem [21,22]. It uses data from different energy spectra during acquisition and the generation of material-specific information. One post-processing application of DECT is to compute virtual monoenergetic images (VMIs), which increase vessel enhancement at low energy levels [23]. Especially small vessels, such as peroneal perforators, may benefit from this technique [24]. Manual assessment of peroneal perforators on standard transverse thin-slice images is time-consuming, given the large number of images and the complex anatomical course of the vessels around the fibular bone. Traditional post-processing methods such as multiplanar reformations (MPRs), maximum intensity projections (MIPs) and 3D volume rendering techniques (VRTs) can help to simplify visualization for the surgeon. The drawback of MIPs and VRTs is a potential loss of information due to their comparatively thick slice volume integration. Therefore, an advanced graphical display that addresses spatial orientation, bone superposition and thin slice information is highly desirable. A novel post-processing algorithm, called Vessel Unfolding Reconstruction (VUR), allows for three-dimensional adjustment of the imaging plane to vessel orientation and allows for the integration of several vessel courses into one single dataset. This reconstruction method may therefore facilitate reliable vessel assessment and comprehensible pre-surgical interdisciplinary planning.

The aim of this study was to evaluate a combination of a dedicated DECT angiography protocol with VMI and VUR reconstructions of the lower limb prior to intended surgical reconstruction of the mandible in comparison to traditional post-processing methods. We hypothesized that VUR enables precise visualization of peroneal perforators and may perform equal to current MPR, MIP and VRT methods.

## 2. Materials and Methods

### 2.1. Patient Population and Study Procedure

Between August 2019 and October 2019, 22 patients underwent DECT angiography for peroneal perforator artery assessment of both lower legs prior to scheduled osteomyocutaneous fibular flap surgery. Exclusion criteria were contraindications for CT examination with intravenous administration of iodine contrast agent, such as grade 4 or higher nephropathy (estimated glomerular filtration rate < 30 mL/min/1.73 m^2^), hyperthyroidism and history of iodine intolerance. All patients signed written informed consent forms. The study protocol was approved by the Institutional Review Board of Friedrich-Alexander Universität Erlangen-Nürnberg (protocol code 14-390 Bn) and adhered to the HIPAA criteria and the Declaration of Helsinki.

### 2.2. Image Acquisition and Image Reconstruction

All CTA examinations were performed on a third-generation dual-source DECT scanner (SOMATOM Force, Siemens Healthineers AG, Forchheim, Germany) using a dedicated examination protocol. A high iodine flux protocol was used for all patients, administering 100 mL of a water-soluble and non-ionic iodine contrast agent intravenously (Imeron 350 mg/mL, Bracco GmbH, Konstanz, Germany). The bolus was injected into a cubital 18 Gauge cannula using a power injector (Accutron CT-D, Medtron AG, Saarbruecken, Germany) at a high flow rate of 7 mL/s, followed by a saline bolus (30 mL, 7 mL/s) after acquisition of the localizer and a premonitoring slice at the level of the knees. A single region of interest (ROI) was placed in one of the popliteal arteries for bolus tracking of the individual scan delay. The threshold was set at 100 Hounsfield Units (HU). Dual-energy acquisition was performed in the cranio-caudal direction using 80 kV for tube A and 150 kV with tin prefiltration (SN) for tube B. The examination range of the lower limb was defined from the P2 segment of the popliteal artery to the calcaneus. A second spiral acquisition was performed immediately after the study sequence in the caudo-cranial direction from the calcaneus to the aortic bifurcation to exclude proximal severe stenosis. This second acquisition was not further evaluated in this study but is part of our clinical routine protocol. Both lower legs of each patient were reconstructed separately in thin slices with overlapping increment and axial orientation (0.6 mm slice thickness, 0.5 mm increment) using a quantitative soft dual-energy kernel. A VMI dataset was calculated in-line on the scanner with a simulated energy of 40 keV (Monoenergetic+, Siemens Healthineers AG, Forchheim, Germany). Advanced iterative reconstruction techniques (ADMIRE, Siemens Healthineers AG, Forchheim, Germany) were used with a blending level of 3. Each field of view was individually adapted to the size of the limb and kept as small as possible (default 140 mm × 140 mm). Detailed scan parameter settings are given in Table 1.

### 2.3. Vessel Segmentation and Post-Processing

Centerlines of the peroneal artery including each of its perforator vessels were automatically defined and manually corrected using the VMI datasets and a dedicated post-processing algorithm (CT Vascular, Syngo.via VB30, Siemens Healthineers AG, Forchheim, Germany) by an experienced radiology resident. Proofreading was independently done by a senior radiologist.

Centerline segmentations were used for curved multiplanar reconstructions (MPRs) for each peroneal perforator vessel. Vessel evaluation was performed in two perpendicular planes in longitudinal axis along the vessel. The position was adjusted at the discretion of the radiologist until the longest possible course was represented.

Maximum intensity projections (MIPs) were reconstructed with 5 mm slice thicknesses and an increment of 1.0 mm over the entire course of the peroneal artery and its perforators. The orientation was adapted to an oblique coronal plane of the posterior peroneal intermuscular septum. 

**Figure 1:**
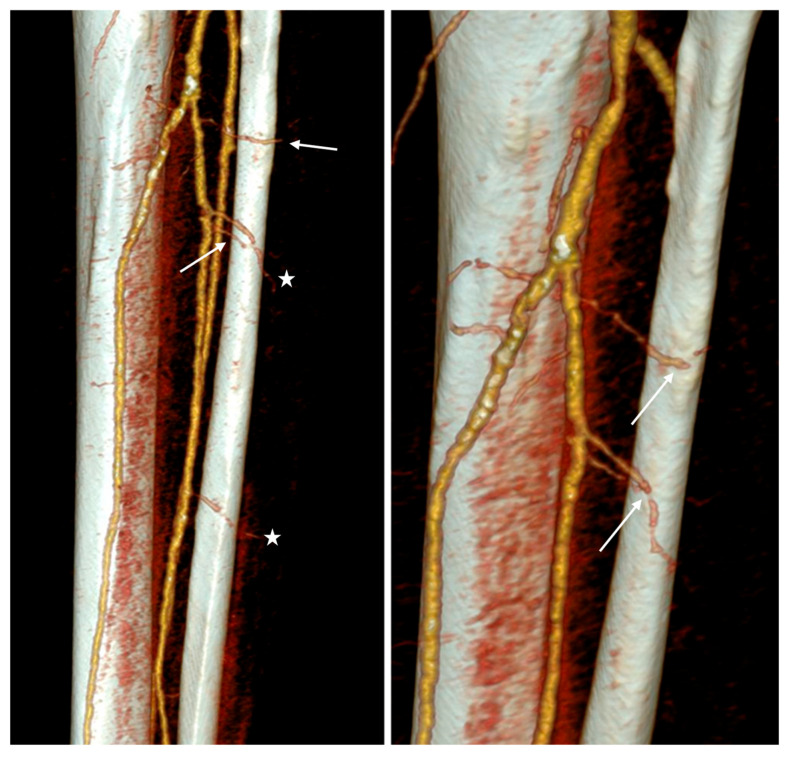
Representative VRT images of one lower leg. **Left side**: Visualization of two peroneal perforator arteries (marked with white asterisks). The proximal vessel and the small vessel below the proximal perforator artery are feeders for the peroneal muscles and do not substantially supply the subcutaneous/cutaneous tissue (marked with white arrows). **Right side**: The same patient with adjusted posterior orientation to highlight the peroneal perforator arteries. White arrows indicate discontinuity of the perforator arteries caused by their contiguity to fibular bone.

Three-dimensional VRT images were reconstructed for each lower limb in a posterior view using the standard parameters for vessel visualization provided by the vendor. Examples are shown in Figure 1.

### 2.4. Vessel Unfolding Reconstruction

**Figure 2:**
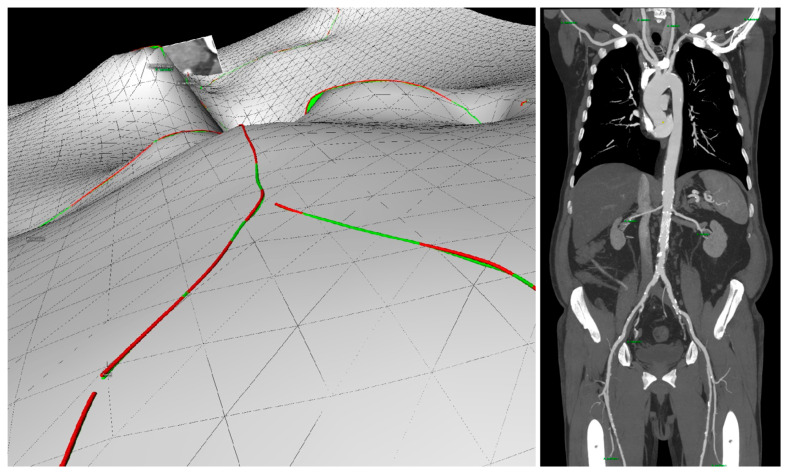
Principle of VUR technique. **Left side**: Centerlines of the aortic vessel tree with its major thoracic and abdominal vessels are drawn in a deformed virtual plane showing the individual complex anatomical course. **Right side**: Aortic and major vessel centerlines are unfolded into the VUR dataset allowing a general overview in a coronal orientation.

**Figure 3:**
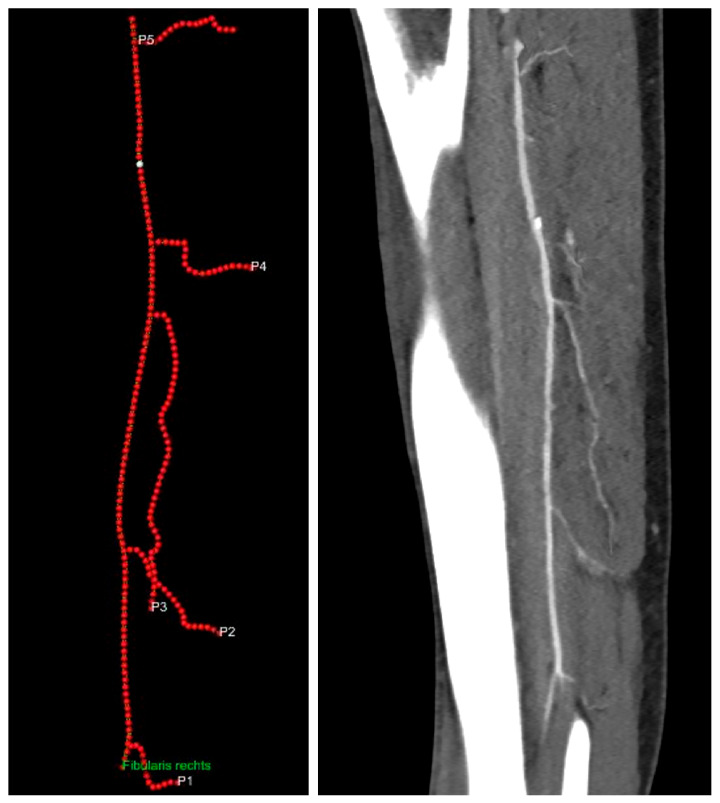
Representative VUR images. **Left side**: Representative image of one patient with five perforator arteries on the right side. The red dotted lines represent the centerlines of fibular artery and its five perforator arteries (P1–P5) visualized as “vessel tree” for general overview. **Right side**: Representative screenshot of the corresponding VUR algorithm derived from the same patient of the right limb side; note that the P4 perforator artery could not be displayed with its full length due to its excessively curved course.

VMIs at 40 keV and centerline segmentations were used as input data for a prototype VUR software algorithm (Siemens Research Frontier, Vessel Unfolding Version 9.9c, Siemens Healthineers AG, Forchheim, Germany). The algorithm merged the individual centerlines together to reconstruct a complete vessel tree per limb. VUR was performed based on this model to produce a single visualization dataset with a three-dimensionally adjusted image plane, in which the complex anatomical courses of different vessels appear as straightened in-plane structures. The grid to voxel factor was set to 8 and the slicing step was 1 mm. The number of slices was set to 20. Image quality was set to the highest quality rendering mode. The principle of this reconstruction technique and a representative example are shown in Figure 2 and Figure 3.

### 2.5. Analysis of Peroneal Perforator Vessels

CTA analysis was performed by two board-certified radiologists using a dedicated server/client-based post-processing software (Syngo.via VB 30, Siemens Healthineers AG, Forchheim, Germany). Both readers were blinded to all patient and other imaging data. The number of perforator vessels was counted for each lower limb using the 40 keV VMIs. In case of discrepancy between both readers, a consensus decision was made and served as gold standard for further analysis. Subsequently, the number of peroneal perforator arteries was independently evaluated for VRTs, oblique coronal MIPs and VUR images in a random order and with a washout period of several days (delay time at least 30 days, maximum 50 days) between each reading session to minimize recall bias from the previous assessment. In case of discrepancy, a consensus decision was reached between both readers. Perforator vessel length was assessed in the curved MPR images, in the oblique coronal MIP images, in the VRT images and in the VUR images using a point-by-point non-linear distance measurement tool in a consensus reading by the above-named investigators.

### 2.6. Statistical Analysis

Interval-level data were evaluated for normal distribution using the Shapiro–Wilk test. Normally distributed data are presented as means ± standard deviation; otherwise non-normally distributed ordinal-level data values are expressed as medians with interquartile ranges. Assuming normal distribution, differences in perforator length values between curved MPR, MIP, VRT and VUR images were evaluated using one-way analysis of variance (ANOVA) with post hoc Holm–Sidak tests for pair-wise comparison. A *p* value < 0.05 was considered statistically significant. Statistical analysis was performed using the software package SPSS Statistics Version 21 (SPSS Inc./IBM, Chicago, IL, USA) and R Statistics (R Core Team; Version 3.6.2; R: a language and environment for statistical computing, R Foundation for Statistical Computing, Vienna, Austria).

## 3. Results

### 3.1. Patient Population

The study population comprised nine female and 13 male patients. The mean age was 59 ± 13 years (range: 31 to 83 years). All 44 examined lower legs were eligible for analysis. On average, CT acquisition time per patient was 3 ± 2 s. The mean CTDI_vol_ was 2.7 ± 0.2 mGy (range: 2.6 to 3.1 mGy). The mean scan range was 365 ± 53 mm, resulting in an average DLP of 136 ± 19 mGy*cm (range: 118 to 173 mGy*cm).

### 3.2. Perforator Quantity Assessment

**Figure 4:**
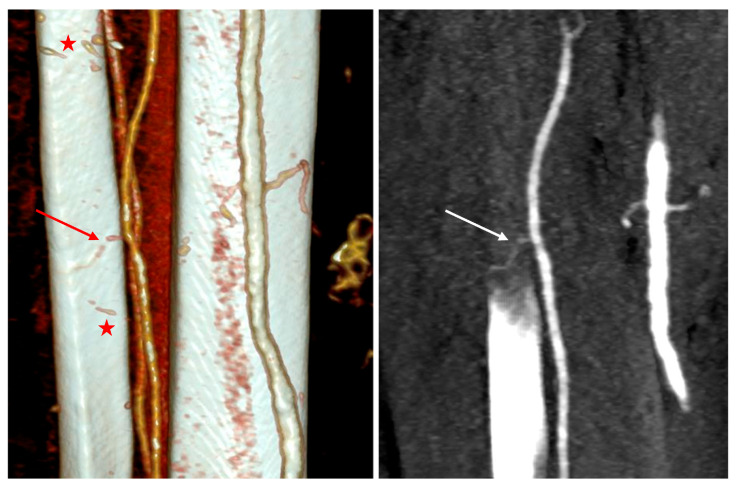
Representative image of one patient with discrepancy in perforator vessel number between VRT and VUR reconstructions. **Right side**: Small perforator vessel could be detected in VUR (white arrow). **Left side**: Hardly visible perforator vessel due to superimposition of the fibular bone, the very small diameter and adjacent similar-appearing soft tissue remnants due to slightly imperfect rendering (red asterisks); both readers did not detect this vessel during assessment (red arrow).

VMIs revealed 113 peroneal perforator vessels in total (mean value 2.6 ± 1.2 per lower leg). The same number was found for oblique coronal MIP and VUR reconstructions. VRT reconstruction showed 109 peroneal perforators (mean value 2.5 ± 1.3 per lower leg). In four patients, one perforator artery could not be discriminated from the adjacent fibular bone in VRT reconstruction (Figure 4 provides an example). 

Perforator vessels were absent bilaterally in one patient in all image reconstructions. In total, 21 patients had at least one perforator vessel on each side. Most patients had 2–3 perforator vessels per limb, a maximum of five perforator vessels per limb was found in one patient on both sides (Figure 3). Table 2 provides a detailed overview of perforator frequencies per lower leg for each reconstruction group.

### 3.3. Perforator Length Assessment

**Figure 5:**
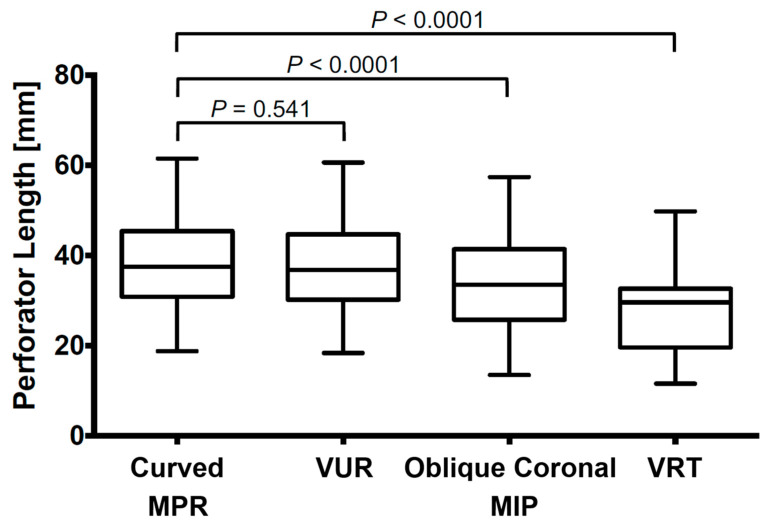
Box plots of peroneal perforator vessel length assessment derived from curved MPR, oblique coronal MIP, VRT and VUR reconstructions.

No significant difference in perforator vessel length was found between curved MPR and VUR (*p* = 0.54), whereas oblique coronal MIP and VRT reconstructions showed significant shorter vessel lengths (both *p* < 0.001) compared to curved MPR. Mean perforator length was 37.2 ± 11.2 mm for curved MPR, 36.7 ± 11.2 mm for VUR, 33.0 ± 11.0 mm for oblique coronal MIP and 27.7 ± 9.8 mm for VRT. Table 3 provides a detailed statistical overview. Box plots are shown in Figure 5.

## 4. Discussion

Low-energetic VMI reconstructions derived from DECT provide feasible vessel contrast in the lower leg, especially for assessment of small arteries such as peroneal perforators. This is valuable for assessing adequate vascular supply and precise vessel localization prior to osteomyocutaneous fibular flap reconstruction. The VUR post-processing algorithm provides a fast, comprehensive overview and detection of peroneal perforators within only a few slices. Compared to single-vessel curved MPR, no discrepancy in number or length was found in this study for the novel VUR technique. Both VRT and oblique coronal MIP reconstructions revealed significantly shorter vessel length values compared to curved MPR and VUR methods. The VRT failed to depict one perforator vessel in four different patients due to superimposition with adjacent fibular bone; in two cases a short segment of the perforator vessel could be identified retrospectively when correlated with the other reconstruction methods. To our knowledge, only few studies are available addressing the evaluation of peroneal perforator arteries with CTA [25,26,27,28,29]. Battaglia et al., studying a comparable patient cohort, reported at least one viable perforator vessel for every lower leg examined [12]. In our study, one patient had no adequate peroneal perforator artery on either side (4.5%), which is in line with the existing literature [30]. Knowledge of absent perforator vessels is essential for the surgeon as it precludes fibular flap harvest and thus avoids unnecessary surgery. Several authors have already advocated preoperative imaging of lower leg vasculature prior to osteomyocutaneous fibular flap transplantation [31,32]. In this context, not only the number and precise location of peroneal perforator arteries are essential, but knowledge of potential vascular anomalies may also have a great impact on surgical planning. Although rare, vascular anomalies such as a dominant peroneal artery or peroneal perforator arteries originating from the posterior tibial artery must be recognized preoperatively to avoid complications and prolonged operative time [33,34,35,36]. In our study cohort, we did not discover any of the above-named vascular anomalies, which is most likely due to our small sample size. Mean number of perforator vessels per lower leg defined by consensus reading in curved MPR was 2.6 in our cohort, which was 23% higher compared to the report from Battaglia et al. (2.1 vessels per lower leg) [12]. However, compared to the aforementioned study, our mean vessel length was much shorter, with an average length of 36 mm vs. 99 mm. This discrepancy may be explained by two factors: Firstly, the CTA acquisition protocols used are substantially different with a distinct venous superimposition in their relatively low-speed acquisition protocol, especially in the subcutaneous section. Secondly, the higher number of detected perforator vessels in our work may partly reflect subtle and shorter vessels that were undetectable with conventional acquisition techniques.

In our opinion, DECT and dedicated post-processing algorithms have the capability to significantly improve identification of vascular composition and possible variants. In particular, the combination of high-contrast VMI reconstructions and comprehensive VUR not only allows for precise anatomical assessment, but also enables simplified and straightforward visualization. The unfolding of the complex three-dimensional architecture of the posterior peroneal septum with imbedded peroneal perforators may serve as a reliable virtual simulation of intraoperative anatomy from the surgeon’s perspective. Beyond perforator detection and length assessment, the functional-anatomical subtype classification of suitable perforators regarding the perforator course from the pedicle to the surface is equally essential for the surgeon [37,38,39]. A commonly used subtype classification in clinical routine comprises (1) pure septocutaneous perforators, (2) septocutaneous perforators with muscular branches, (3) perforators with muscular branches only, and (4) indeterminate perforators due to impaired visualization [18,39]. In a recent study by our group, perforator detection rates were fully comparable between a time-of-flight (TOF) acquisition protocol with ultra-high-field MRI (7 Tesla; 7 T) and a dedicated dual-energy CTA scanner using similar low-energy reconstruction parameters as applied in the present study. Notably, both perforator length assessment and subtype classification were significantly superior with TOF-MRA [18]. This finding can be attributed to several factors: Ultra-high-field MRI is capable of reaching the highest possible spatial resolution available for in vivo studies using MRI and therefore allows precise depiction of even small perforators. Furthermore, compared to CT, MRI offers a higher inherent soft tissue contrast, which is especially beneficial for peroneal perforator subtype classification. In contrast, 7 T MRI is costly and prone to motion artifacts due to long acquisition times using TOF methods, whereas CTA can be performed in a matter of seconds as part of routine clinical workup. Furthermore, 7 T technology is limited to a few dedicated centers and is not comprehensively available. A study evaluating CTA vs. MRA for peroneal perforator assessment using lower field strengths is still missing to date. Future comparative studies should evaluate peroneal perforator assessment between, for example, 3 T MRA and dual-energy CTA with the VUR algorithm, not only regarding perforator detection and length assessment, but also the feasibility of functional-anatomical subtype classification. Another X-ray-based modality applicable to peroneal perforator diagnostics is conventional invasive angiography, which is also capable of depicting small and very small vessels and was historically the method of choice [10,40,41,42]. A specific disadvantage of this method is its invasiveness, carrying a risk of bleeding and infection. A further limitation concerns the procedure itself: conventional angiography typically evaluates only the potential donor limb via a single femoral arterial access, thereby restricting peroneal perforator assessment to one side. In contrast, CTA and MRA allow simultaneous evaluation of both lower limbs, which may provide valuable information from the contralateral side, particularly when the designated donor limb lacks suitable perforators. Dedicated 3D acquisition techniques (“Cone Beam Computed Tomography”; CBCT) are available in the angiography suite for subtype classification in certain centers, but the soft tissue contrast delivered by CBCT is typically inferior to conventional CTA reconstructions due to increased X-ray scattering and limited sensitivity for small density differences, thus potentially limiting the subtype classification [43,44]. Using basic conventional two-dimensional angiography images (“Digital Subtraction Angiography”; DSA), perforator subtype classification is almost impossible, which is a major drawback of this method. Ultrasound examination is widely used by clinicians, who specifically use color-coded or basic Doppler mode to assess peroneal perforators [45]. Notable advantages include the absence of ionizing radiation, straightforward accessibility and applicability, high cost-effectiveness, and the obviation of intravenous or intra-arterial contrast agent administration. In addition, ultrasound examination is also feasible under sterile conditions during surgery, and can directly guide the surgeon to the desired perforator vessel. However, the modality is very user-dependent and its penetration depth varies depending on the specific device and probe frequency, favoring superficial structures. Deeper compartments, e.g., iliac arteries, may be difficult to assess for potential stenosis, which in turn may limit surgical success. In a recent study by Walczak et al., color-coded Doppler showed superior performance over handheld Doppler for preoperative fibular flap design [46]. In a meta-analysis by Moore et al., color-coded ultrasound was shown to provide reliable preoperative sensitivity compared to CTA regarding preoperative perforator assessment for Anterolateral Thigh Flap (ALT) surgery, whereas to the best of our knowledge no study exists comparing the diagnostic performance between ultrasound, CTA and MRA regarding peroneal perforator assessment [47]. As outlined above, a structured comparison of DECT with a VUR algorithm and color-coded ultrasound in future studies would be of considerable interest regarding the diagnostic performance of each modality, particularly regarding intraoperative correlation. Table 4 provides an overview of available imaging strategies for peroneal perforator diagnostics.

In conclusion, high-resolution DECT and VUR enable precise preoperative vessel detection and length assessment and may be regarded as a feasible alternative compared to traditional post-processing visualization methods. However, compared to other modalities such as MRA or ultrasound, future studies are warranted to compare the diagnostic performance of VUR, particularly with regard to the pre-surgical functional-anatomical subtype classification of peroneal perforators. VRT reconstruction demonstrates significant limitations in peroneal perforator detection and vessel length assessment and therefore should be avoided for pre-surgical perforator diagnostics. Moreover, the improved visualization with VUR may not only enable a reliable diagnosis by the radiologist, but also facilitate communication with the treating surgeon, enhance interdisciplinary collaboration and potentially improve intraoperative correlation with the preoperative vessel assessment.

Some limitations of our study must be acknowledged. Firstly, the number of study participants in this retrospective single-center study included was limited, but 44 lower legs assessed may be considered suitable for a pilot study demonstrating the feasibility of the VUR technique. Secondly, we are unable to provide intraoperative correlation as the gold standard for all cases, because we evaluated both sides of each patient and five patients were considered unsuitable for fibular transplantation. Moreover, other modalities such as Magnetic Resonance Imaging, conventional invasive angiography and ultrasound examination were not considered in this study. Thirdly, our analysis is limited to the lowest available VMI reconstruction; this study design does not allow conclusions to be drawn regarding the optimal trade-off between image artifacts and vascular contrast using different keV levels. Fourthly, the feasibility of functional-anatomical subtype classification was not assessed across the compared post-processing methods.

## 5. Conclusions

The combination of low-energetic VMI reconstructions derived from DECT acquisition and a VUR post-processing algorithm is feasible for the evaluation of peroneal perforator arteries prior to osteomyocutaneous fibular flap reconstruction.

## Figures and Tables

**Table 1:** Scan parameters and image reconstruction settings.

Scan Parameter	Setting/Value
Tube Voltage (kV)	Tube A: 80 Tube B: 150
Tube Current (mAs)	120
Pitch	1.0
Reconstruction Kernel VMI	Bv36d
In-Line VMI Reconstruction (keV)	40
Collimation (mm)	128 × 0.6
VMI Reconstruction Slice Thickness (mm)	0.6 (Increment 0.5)
Iterative Reconstruction	ADMIRE Level 3
Rotation Time (s)	0.25
CTDIvol (mGy; mean ± standard deviation)	2.5 ± 0.4
Duration (s; mean ± standard deviation)	3 ± 2
Default Scan Range Z-Axis (mm)	352

**Table 2:** Frequency of detected vessel quantity per lower leg in the different reconstruction series (in 44 total analyzed lower legs).

Number of Perforator Vessel(s) per Lower Leg	VMI	Oblique Sagittal MIP	VRT	VUR
0	2	2	2	2
1	8	8	9	8
2	9	9	11	9
3	15	15	12	15
4	8	8	8	8
5	2	2	2	2
Total sum of detected perforators	113	113	109	113

**Table 3:** Peroneal perforator length assessment for curved MPR, oblique-coronal MIP, VRT and VUR.

	Mean Vessel Length	Standard Deviation	Maximum	Minimum
Curved MPR	37.2 mm	11.2 mm	61.5 mm	18.8 mm
Oblique coronal MIP	33.0 mm	11.0 mm	57.4 mm	13.5 mm
VRT	27.7 mm	9.8 mm	49.8 mm	11.6 mm
VUR	36.6 mm	11.2 mm	60.6 mm	18.4 mm

**Table 4:** Overview of different imaging strategies for peroneal perforator arteries, highlighting specific diagnostic feasibility and potential limitations. PPA: Peroneal perforator artery.

Modality	Conventional CTA	Vessel Unfolding Reconstruction (VUR) with Dual-Energy Acquisition	Magnetic Resonance Angiography (MRA)	Invasive Angiography	Ultrasound Examination
Availability and ease of execution	Comprehensively available	Limited to certain centers	Comprehensively available (1.5 & 3 Tesla) 7 Tesla limited to specialized centers	Comprehensively available	Comprehensively available
Ability to asses major vessel patency and possible anomalies (A. peronea)	Sufficient vessel diagnostics	Sufficient vessel diagnostics with enhanced iodine contrast	Sufficient vessel diagnostics	Sufficient vessel diagnostics, usually limited to one side	Sufficient vessel diagnostics of superficial vessels
Ability to asses small peroneal perforators	Assessment limited, mainly due to poor vessel contrast	Assessment improved due to enhanced iodine contrast	Assessment feasible; spatial resolution reduced at low field strength	Assessment feasible, esp. with catheter placed in peroneal artery	Small perforators detectable in color and Doppler mode
Potential limitations	Functional-anatomical PPA classification challenging Iodine contrast agent mandatory	Functional-anatomical PPA classification may benefit from enhanced iodine contrast Iodine contrast agent mandatory	Ferromagnetic substances may prohibit examination Time-consuming	Functional-anatomical PPA classification not feasible due to two-dimensional acquisition Three-dimensional acquisition limited to certain centers Higher risk of complications due to invasive procedure Contrast agent mandatory	User dependency Deeper vessel compartments difficult to assess
Costs	Cost-effective	More expensive due to high purchase price	Expensive due to high purchase price (esp. high-field MRI) and comparatively time-consuming examination time	Expensive due to invasive character and necessary recovery time after femoral puncture	Very cost-effective

## Data Availability

All data generated and analyzed during this study are included in this published article. Raw data supporting the findings of this study are available from the corresponding author on request.

## Abbreviations

The following abbreviations are used in this manuscript:

CTA	Computed Tomography Angiography
VUR	Vessel Unfolding Reconstruction
MPR	Multiplanar Reformation
MIP	Maximum Intensity Projection
VRT	Virtual Rendering Technique
DECT	Dual-Energy Computed Tomography
VMI	Virtual Monoenergetic Image
ROI	Region of Interest
SN	Tin Prefiltration
HU	Hounsfield Unit
keV	Kiloelectron Volt
kV	Kilovolt
mAs	Milliampere-Second
mGy	Milligray
S	Second
CTDI_vol_	Volume Computed Tomography Dose Index
DLP	Dose Length Product
